# Comprehensive compensation of real-world degradations for robust single-pixel imaging

**DOI:** 10.1038/s41377-025-02021-7

**Published:** 2025-10-13

**Authors:** Zonghao Liu, Bohan Yang, Yifei Zhang, Junfei Shen, Xin Yuan, Mu Ku Chen, Fei Liu, Zihan Geng

**Affiliations:** 1 Tsinghua Shenzhen International Graduate School, Tsinghua University, Shenzhen, China; 2 Pengcheng Laboratory, Shenzhen, China; 3 College of Electronics and Information Engineering, Sichuan University, Chengdu, China; 4 AI Department, School of Engineering, Westlake University, Hangzhou, China; 5 Department of Electrical Engineering, City University of Hong Kong, Kowloon, Hong Kong SAR China; 6 School of Optoelectronic Engineering, Xidian University, Xi’an, China

**Keywords:** Imaging and sensing, Applied optics

## Abstract

Single-pixel imaging (SPI) faces significant challenges in reconstructing high-quality images under complex real-world degradation conditions. This paper presents an innovative degradation model for the physical processes in SPI, providing the first comprehensive and quantitative analysis of various SPI noise sources encountered in real-world applications. Especially, pattern-dependent global noise propagation and object jitter modelling methods for SPI are proposed. Subsequently, a deep-blind neural network is developed to remove the necessity of obtaining parameters of all the degradation factors in real-world image compensation. Our method can operate without degradation parameters and significantly improve the resolution and fidelity of SPI image reconstruction. The deep-blind network training is guided by the proposed comprehensive SPI degradation model that describes real-world SPI impairments, enabling the network to generalize across a wide range of degradation combinations. The experiment validates its advanced performance in real-world SPI imaging at ultra-low sampling rates. The proposed method holds great potential for applications in remote sensing, biomedical imaging, and privacy-preserving surveillance.

## Abstract (web summary)

This paper presents a comprehensive and quantitative analysis of noise sources in real-world single-pixel imaging systems. Based on the proposed physical model, a robust and practical compensation network is developed.

## Graphical abstract

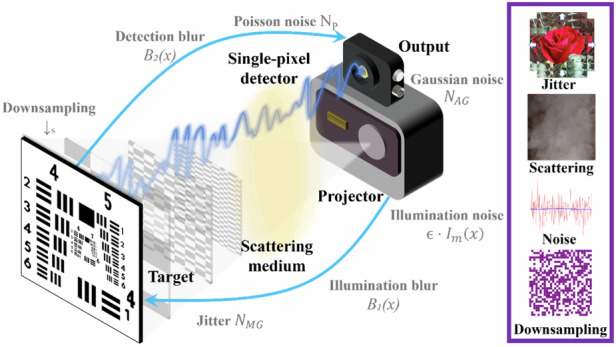

## Introduction

Single-pixel imaging (SPI) is an innovative and cost-effective imaging technology that leverages a single-pixel detector and specific light field modulation patterns to capture spatial information and reconstruct images^1–3^. This technique is especially applicable in conditions where the use of pixel array detectors becomes impractical due to their high costs or challenges in fabrication, particularly for certain non-visible light wavebands^4^. On the other hand, in conditions of low light intensity, where pixel array detectors struggle to perform well, SPI demonstrates significant advantages, providing a superior solution compared to traditional imaging methodologies^5^. It has been applied in various fields and demonstrated unique advantages, including image compressive sensing^6,7^, remote sensing^8^, three-dimensional imaging^9^, privacy protection^10^, moving object trajectory tracking^11,12^, and multispectral imaging^13,14^.

The role of a degradation model in image recovery is of paramount importance^15^. Creating a comprehensive model that closely mimics the actual circumstances can lead to remarkable improvements in image quality^16^. However, the degradation model for SPI is markedly different from that of a conventional pixel array camera. Compared with conventional imaging, SPI includes distinct operations, such as coded illumination modulation, intensity integration across the entire scene and computational image reconstruction. The detailed comparison of the degradation processes between the conventional passive imaging and SPI is provided in Supplementary Section 1 and Fig. S1.

Some studies are conducted for SPI degradation modeling. For instance, in 2017, Lyu et al.^17^ proposed a neural network that maps noisy computational ghost imaging (CGI) reconstructions to high-quality images. However, this method lacks generalization to different degradation types. In 2021, Hu et al.^18^ developed a generative adversarial network (GAN) to enhance Fourier SPI degraded by frequency truncation at low sampling ratios. This method reduces the ringing artefacts in the reconstructed images. In many real-world scenarios, the imaged objects are not perfectly static. Due to the sequential acquisition process of SPI, even minor temporal fluctuations—such as object motion, platform jitter, or illumination instability—can cause significant inconsistencies across measurements. This introduces complex and global artefacts in the reconstructed image, highlighting the need for a degradation model that faithfully captures the temporal sensitivity of SPI systems. In 2020, Huang et al. investigated temporal fluctuations in SPI caused by object or platform trembling^19^. They propose to treat the movement process as a number of intermediate static states. However, it requires extensive computation. Scattering-like degradations—such as haze, fog, optical defocus, and mild underwater turbidity—are ubiquitous in real-world environments and can profoundly impact the effectiveness of SPI applications. Le et al.^20^ conducted underwater SPI to examine the robustness of CGI reconstruction under scattering and attenuation. Huang et al.^21^ extended their analysis to wind conditions by modeling the optical wavefront with airflow-induced phase perturbations. While some degradation factors of SPI imaging have been studied, pattern-dependent global noise propagation process and object jitter modeling methods remain to be further investigated. More importantly, most of the existing degradation compensation methods only compensate for one degradation factor and require accurate degradation parameters. A comprehensive study and a feasible solution for complex real-world degradations are needed in the SPI field.

This study addresses the challenge of robust reconstruction in SPI under complex and unknown degradations by proposing a pair of physically grounded and generalizable degradation modeling and compensation approaches. A comprehensive physical degradation model and two analytic approaches are developed, including: (1) a pattern-dependent global noise propagation mechanism, which describes how the local measurement noise in SPI propagates globally in the reconstructed image; and (2) a boundary-aware multiplicative noise model, which simulates jitter-induced signal instability in SPI imaging. This physical model enables the learning of a deep-blind reconstruction network that performs robustly under real-world SPI degradations and low sampling rates. The proposed approach is compatible with various structured illumination schemes, including Hadamard and Fourier-based SPI^22,23^. It is applicable to diverse domains such as remote sensing, biomedical imaging, and privacy-preserving surveillance.

## Results

### Structured degradation modeling and blind compensation architecture

**Fig. 1:**
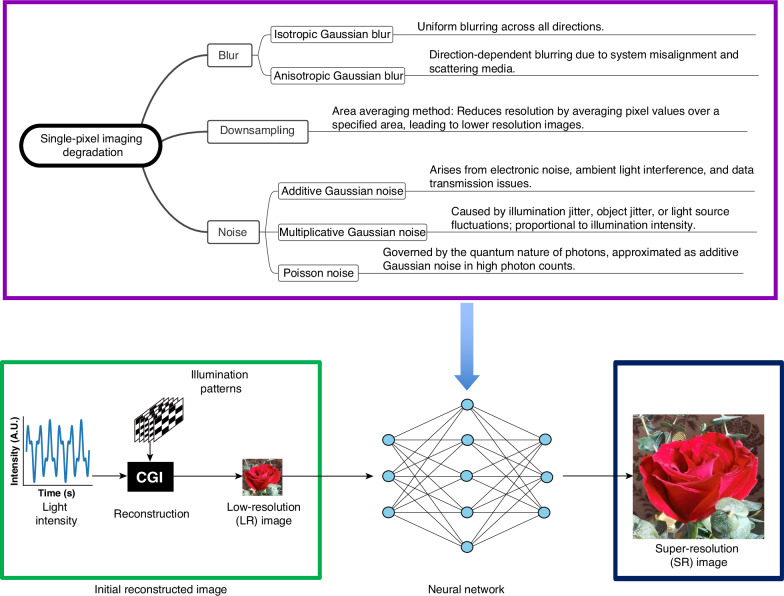
**Overview of the proposed framework.** A comprehensive degradation model is studied for a variety of real-world image impairments. The reconstruction process begins with computational ghost imaging (CGI) applied to single-pixel measurements, yielding a coarse, low-resolution image. A neural network trained based on the degradation physical models restores and generates high-resolution images

As depicted in Fig. 1, an analytic and comprehensive degradation model is developed to train a deep-blind degradation compensation neural network that can compensate for different types of degradations at the same time. The object is illuminated by a series of Hadamard patterns, and the corresponding reflected intensities are captured by a single-pixel detector. Then, the object image is reconstructed by correlating the known illumination patterns with the measured intensities through CGI^24^. Details of the CGI algorithm are shown in Supplementary Section 2. After low-resolution image reconstruction via the CGI method, the proposed neural network, named Blind Super-Resolution Single-Pixel Imaging (BSRSPI), compensates for complex real-world degradations and improves the image resolution.

**Fig. 2:**
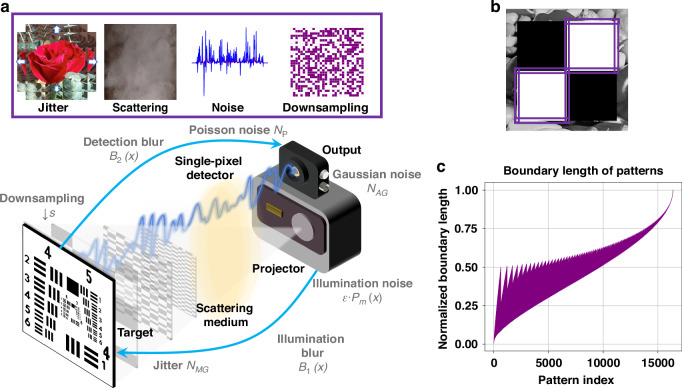
**Degradation modeling and pattern boundary analysis in single-pixel imaging.** **a** Schematic illustration of the single-pixel imaging system with key degradation sources. **b** Schematic visualization of the boundaries of the binary illumination patterns. The purple boxes mark the regions sensitive to spatial jitter during data acquisition. **c** Normalized boundary length of 16,384 Hadamard patterns, defined as the total length of bright–dark transition edges in each binary pattern. Patterns with longer illuminated boundaries are more susceptible to spatial jitters during acquisition, as reflected in the corresponding variance of the multiplicative Gaussian noise model

The degradation process of SPI is indicated in Fig. 2a. The illumination patterns from the projector undergo scattering and non-ideal focus, introducing blur during the illumination stage. The modulated light pattern is projected onto the object, where spatial downsampling occurs due to the limited resolution of the patterns. Mechanical jitters between the object and the projection system during acquisition introduce relative misalignment, leading to multiplicative fluctuations in the measurement. After modulation, the reflected light may experience additional degradation along the detection path due to scattering imperfections, resulting in further blur. Finally, the photon shot noise and the electronic noise affect the detection process. The photon shot noise is modeled as a Poisson-distributed function due to the quantum nature of light.

The forward measurement of SPI under illumination pattern ${P}_{m}\left(x\right)$ is modeled as:

1 $${D}_{m}=\left(\int { {\mathcal B} }_{2}\left({ {\mathcal B} }_{1}\left({\varepsilon }_{\mathrm{illum},m}\cdot {P}_{m}\left(x\right)\right)\cdot {\downarrow }_{s}(GT\left(x\right))\right)\cdot dx\right)\cdot (1+{\delta }_{m})+{\varepsilon }_{\mathrm{add},m}$$

Here:

- $x$ is the location of each pixel;
- ${GT}\left(x\right)$: Ground-truth reflectance or transmittance from the scene;
- ${{\rm{\varepsilon }}}_{\mathrm{illum},m}\sim {\mathcal{N}}(1,{\sigma }_{\text{illum}}^{2})$: Multiplicative Gaussian noise due to illumination fluctuations;
- ${{\mathcal{B}}}_{1}\left(\cdot \right)$: Blur kernel of illumination-path degradations (e.g., scattering, defocus);
- ${\downarrow }_{s}\left(\cdot \right)$: Downsampling of factor $s$;
- ${{\mathcal{B}}}_{2}\left(\cdot \right)$: Blur kernel of detection-path degradations (primarily scattering);
- ${P}_{m}\left(x\right)$: *m*th illumination pattern;
- ${D}_{m}$: Measurement result of the *m*th pattern;
- ${\delta }_{m}\sim {\mathcal{N}}\left(0,{\sigma }_{\text{jitter},m}^{2}\right)$: Multiplicative Gaussian noise due to object or pattern jitter for the *m*th pattern;
- ${\varepsilon }_{\mathrm{add},m}\sim {\mathcal{N}}\left(0,{\sigma }_{{\rm{P}},m}^{2}+{\sigma }_{\mathrm{AG}}^{2}\right)$: Combined additive Gaussian noise, where ${\sigma }_{{\rm{P}},m}^{2}$ approximates the variance of Poisson-distributed photon noise for the *m*th measurement. When the number of detected photons is sufficiently large, Poisson noise can be approximated by a signal-dependent Gaussian distribution with variance proportional to the signal intensity. The second term ${\sigma }_{{\rm{AG}}}^{2}$ represents the variance of signal-independent additive Gaussian noise.

*The detailed analysis of each degradation factor is listed in the following sections*.

#### Noise

As an active imaging procedure, the sources of noise in SPI are more diverse than in conventional passive imaging due to active illumination. The degradation types and orders are different. Another key difference lies in the error propagation procedure. In conventional imaging, additive noise typically affects each pixel within a small local region. In SPI, as the single-pixel detector integrates the collected light intensities from the entire scene, any noise from each photodetector readout can propagate and spread to the entire image after reconstruction. This section elaborates on noise factors in SPI, such as multiplicative Gaussian noise, additive Gaussian noise, Poisson noise, etc.

##### Multiplicative Gaussian noise (MGN) N_MG_

During the process of SPI, random jitters from the object or the illumination platform can degrade the quality of image reconstruction^19^. As this degradation is directly proportional to the illumination intensity, it is modeled as a MGN

2 $${D}_{m}={S}_{m}\times (1+{\delta }_{m})$$

Here, ${S}_{m}$ represents the original signal for the *m*-th pattern. ${D}_{m}$ denotes the resulting signal for that pattern. ${\delta }_{m}$ signifies the MGN. This noise is characterized as a Gaussian random process with a zero mean and a standard deviation of ${\sigma }_{\text{jitter},m}$. The probability density function (PDF) of MGN is given by:

3 $$P\left({\delta }_{m}\right)=\frac{1}{\sqrt{2\pi }{\sigma }_{\text{jitter},m}}\cdot {e}^{-\frac{{{\delta }_{m}}^{2}}{2{\sigma }_{\text{jitter},m}^{2}}}$$

Here, ${\delta }_{m}$ denotes the multiplicative Gaussian noise for the *m*th pattern. It can be discerned from Fig. 2b, the jitter noise primarily emanates from the illuminated boundaries of the projection patterns. The longer these lighting boundaries are, the more noticeable the noise becomes. To describe this relationship quantitatively, the noise variance ${\sigma }_{\text{jitter},m}^{2}$ is defined as a term proportional to the boundary length of the *m*th illumination pattern:

4 $${\sigma }_{\text{jitter},m}^{2}=\alpha \cdot {B}_{m}$$

where ${B}_{m}$ denotes the normalized boundary length of the *m*th pattern and $\alpha$ is a global scaling coefficient. In this study, the illuminated boundary lengths are taken from the 128 × 128 cake-cutting Hadamard illumination patterns^25^ employed in the experiments. The results are depicted in Fig. 2c, where the *x* axis signifies the order of the projection patterns, and the y-axis indicates the length of the illuminated boundaries.

In addition to the jitter-induced multiplicative Gaussian noise discussed above, the light source itself may introduce fluctuations in illumination intensity due to electrical instability or temporal flicker. These fluctuations manifest as multiplicative noise applied before projection and are independent of the object or pattern structure. This is modeled as a scalar multiplicative perturbation ${\varepsilon }_{\mathrm{illum},m}\sim {\mathcal{N}}\left(1,{{\sigma }_{\mathrm{illum}}}^{2}\right)$, where ${{\sigma }_{{\rm{illum}}}}^{2}$ reflects the fluctuation level of the illumination source. This noise acts globally across the entire field of view, modifying the projected pattern intensity ${P}_{m}\left(x\right)$ as:

5 $${P}_{m}^{\text{actual}}\left(x\right)={\varepsilon }_{{\rm{illum}},m}\cdot {P}_{m}\left(x\right)$$

##### Additive Gaussian noise (AGN) N_AG_

In SPI systems, additive Gaussian white noise can arise at various stages of the signal acquisition pipeline. Major sources include readout noise from the photodetector and analog front-end circuitry (e.g., charge-to-voltage conversion, amplifier noise, analog-to-digital converter quantization), noise due to ambient light interference or background leakage, thermal noise and electronic fluctuations in power and amplification circuits. The distribution of this noise can be expressed as

6 $$P\left({N}_{\mathrm{AG}}\right)=\frac{1}{\sqrt{2\pi }{\sigma }_{\mathrm{AG}}}\cdot {e}^{-\frac{{{N}_{\mathrm{AG}}}^{2}}{2{{\sigma }_{\mathrm{AG}}}^{2}}}$$

Here, *N*_AG_ denotes the AGN, while ${\sigma }_{{\rm{AG}}}$ represents its standard deviation^26^.

##### Poisson noise (PN) N_P_

In single-pixel imaging, the capture of photons by the detector is governed by the quantum nature of photons, making their arrival a stochastic event that follows a Poisson distribution. When the number of photons is sufficiently large, the photon statistical noise can be approximated as additive Gaussian noise $\omega {\sigma }_{m}{\varepsilon }_{m}$:

7 $${D}_{m}=\int {P}_{m}(x)GT(x)dx+\omega {\sigma }_{m}{\varepsilon }_{m}$$

In this model, ${D}_{m}$ is the measured signal corresponding to the *m*th illumination pattern. $x$ is the location of each pixel. ${P}_{m}\left(x\right)$ denotes the intensity distribution of the *m*th pattern. ${GT}\left(x\right)$ is the ground-truth reflectance or transmittance from the scene. The noise term is expressed as ${\sigma }_{m}{\varepsilon }_{m}$, where ${\varepsilon }_{m}\sim {\mathcal{N}}\left(\mathrm{0,1}\right)$ denotes standard Gaussian white noise. ${\sigma }_{m}$ denotes the baseline standard deviation of photon noise, which is proportional to the square root of the signal intensity, in accordance with Poisson statistics. The scalar $\omega$ represents the overall noise level or system-specific noise scaling factor^17,27^.

The variance of the noise term is given by:

8 $${{\sigma }_{P}}^{2}={{\rm{\omega }}}^{2}{{\rm{\sigma }}}_{m}^{2}={{\rm{\omega }}}^{2}\int {P}_{m}(x)\cdot GT(x)dx$$

In this formulation, the Poisson-distributed photon noise is approximated by a Gaussian distribution with a signal-dependent variance. This approximation is valid in the high-photon regime, where the Poisson distribution converges toward a Gaussian according to the central limit theorem. Despite being signal-dependent, the additive form of this noise allows it to be jointly modeled with signal-independent Gaussian noise in a unified formulation. The combined variance of these additive Gaussian components is given by:

9 $${\sigma }_{{\text{add}},m}^{2}={\sigma }_{{\rm{P}},m}^{2}+{\sigma }_{\text{AG}}^{2}$$

where ${\sigma }_{{\rm{P}}}^{2}$ denotes the photon noise variance, and ${\sigma }_{\text{AG}}^{2}$ represents signal-independent additive Gaussian noise.

#### Downsampling

In SPI, each measurement intrinsically corresponds to the spatial integration of light reflected or transmitted from the scene modulated by the projected pattern. Therefore, to maintain physical consistency with the data acquisition process, downsampling is modeled as a uniform averaging operation in the high-resolution (HR) image.

Let ${I}_{{\rm{HR}}}$ denote a high-resolution image of size *M* *×* *M*, and ${I}_{{\rm{LR}}}$ the corresponding low-resolution image of size *N* *×* *N*, where *M* = *kN* and *k* is an integer downsampling factor. Under this model, each pixel in the low-resolution image is computed as the average of a *k* *×* *k* block in the HR image:

10 $${I}_{\mathrm{LR}}\left(i,j\right)=\frac{1}{{k}^{2}}\mathop{\sum }\limits_{a\,=\,{ki}}^{{ki}+k-1}\mathop{\sum }\limits_{b\,=\,{kj}}^{{kj}+k-1}{I}_{\mathrm{HR}}\left(a,b\right)$$

for $i,j$ = 0,1,…,*N* − 1. Here, $i,j$ represent the row and column indices of pixels in the low-resolution image ${I}_{{\rm{LR}}}$. $a,b$ denote the row and column indices of pixels in the high-resolution image ${I}_{{\rm{HR}}}$. This formulation ensures that the simulated downsampling process faithfully reflects the spatial integration nature of SPI measurements.

#### Blur

In prior studies, SPI blur is often modeled by an isotropic Gaussian blur kernel^28^, which effectively approximates uniform defocus and symmetric scattering effects—such as those induced by thin mist layers or ground-glass diffusers. However, in practical optical systems, various factors—such as mild lens misalignment, directional defocus, and the anisotropic distribution or geometry of scattering media—can introduce direction-dependent blurring^29^. As such, it is necessary to incorporate anisotropic blur kernels into the degradation model.

In the context of this study, two distinct Gaussian blurring processes are incorporated. The first employs an isotropic Gaussian kernel, denoted as *B*_iso_, which assumes uniform blurring in all directions. The second, on the other hand, utilizes an anisotropic Gaussian kernel, represented as *B*_aniso_^30^. This anisotropic kernel accounts for variable blurring across different directions, providing a more intricate and possibly more accurate representation of real-world optical degradations than isotropic modeling. Their formulations are as follows,

11 $${B}_{{\rm{iso}}}\left(x,y\right)=\frac{1}{2\pi {{\sigma }_{{\rm{iso}}}}^{2}}{e}^{-\frac{{x}^{2}+{y}^{2}}{2{{\sigma }_{{\rm{iso}}}}^{2}}}$$

12 $${B}_{{\rm{aniso}}}\left(x,y\right)=\frac{1}{2\pi {\sigma }_{x}{\sigma }_{y}}{e}^{-\frac{{x}^{2}}{2{{\sigma }_{x}}^{2}}-\frac{{y}^{2}}{2{{\sigma }_{y}}^{2}}}$$

For the isotropic Gaussian kernel, ${\sigma }_{{\rm{iso}}}$ represents the standard deviation. In the case of the anisotropic Gaussian kernel, ${\sigma }_{x}$ and ${\sigma }_{y}$ denote the standard deviations in the *x* and *y* directions, respectively. The variables *x* and *y* indicate the horizontal and vertical distances from the center of the kernel.

#### Network structure

To address the challenges of signal-dependent global degradation in SPI, a GAN-based super-resolution architecture is constructed, comprising a generator and a multi-scale discriminator. The generator employs a densely connected residual backbone and progressive upsampling to recover high-resolution images from CGI inputs (LR image). The discriminator has hierarchical downsampling and skip-connected upsampling to achieve both global consistency and local texture fidelity.

The network is trained under a composite loss function that combines pixel-wise image loss, adversarial loss, and perceptual loss. While L1 loss enforces pixel-level fidelity, it often leads to over-smoothed results under blind or severe degradations. To overcome this problem, a perceptual loss computed in the Visual Geometry Group^31^ feature space is introduced to encourage visually realistic textures and high-frequency details. The adversarial loss further regularizes the output distribution toward natural image statistics. More details of our network are provided in Supplementary Section 3 and Fig. S2.

### Simulation results

To evaluate the robustness of the proposed degradation compensation framework under complex, unfavorable conditions, simulation experiments are conducted on various representative degradations commonly found in SPI, including (1) Blur, including isotropic and anisotropic Gaussian kernels, with varying standard deviations to simulate different degrees of defocus and scattering; (2) Noise, encompassing both additive and multiplicative types, further categorized into pattern-dependent (e.g., signal-dependent Poisson noise and jitter-induced multiplicative noise) and pattern-independent (e.g., sensor readout noise and illumination fluctuations). Each noise source is sampled with a variance drawn from a predefined interval; (3) Sampling ratios, which are randomly selected within the range of 5.0% to 6.25% to simulate ultra-low acquisition conditions. This structured stochastic degradation strategy allows the network to experience a broad distribution of degradation conditions during training, thereby enhancing its generalization to blind and real-world SPI scenarios. The details of data generation are provided in Supplementary Section 4. The definitions of degradation levels are provided in Supplementary Section 5.

The proposed method is compared against several state-of-the-art approaches that incorporate recent deep learning technologies, including diffusion models, dual-branch CNNs, and adversarial frameworks. Specifically, DDPMGI (Denoising Diffusion Probabilistic Model Ghost Imaging)^32^, PCM-DRGI (Photon Contribution Model-based Degradation-Guided Ghost Imaging)^33^, and GAN-SRSPI (GAN-based Super-Resolution Single-Pixel Imaging)^34^ are selected as representative baselines. A combination of the DIV2K^35^ dataset (900 images) and the Flickr2K^36^ dataset (2650 images) is used for comparison, resulting in a total of 3550 diverse high-resolution images for model training in single-image super-resolution tasks. To avoid overfitting and ensure reliable evaluation, 10% of the images (355) are reserved as a held-out test set. The synthesized measurements are generated using the proposed physically grounded SPI degradation model.

**Fig. 3:**
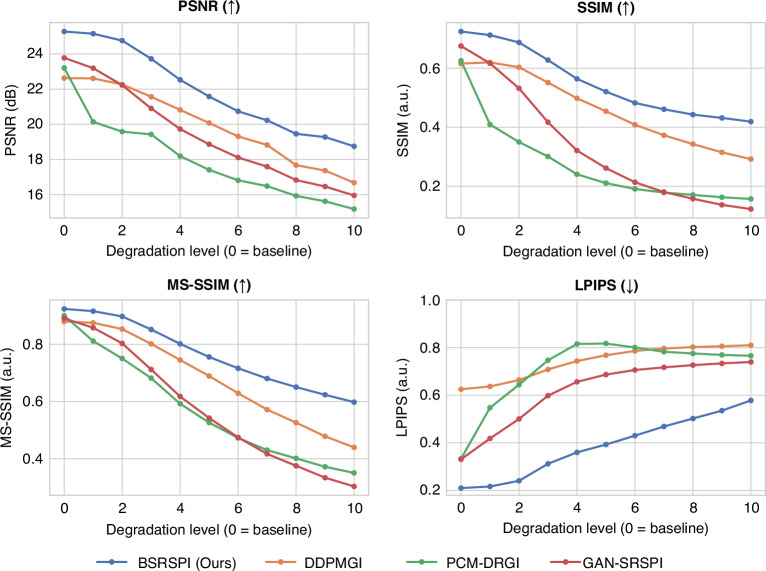
**Quantitative evaluation under different levels of composite degradation, including multiplicative illumination noise, spatial blur (isotropic and anisotropic), random jitter, and additive detection noise (Poisson and Gaussian).** The comparison includes PCM-DRGI^33^, DDPMGI^32^, GAN-SRSPI^34^, and the proposed BSRSPI. Across all metrics, the proposed BSRSPI consistently outperforms competing methods, demonstrating superior robustness in real-world scenarios. Arrows (↑) indicate that a higher value is better

The SPI imaging results comparison under the above-mentioned comprehensive degradations are presented in Fig. 3. More details of the degradation composition and the SPI imaging results under each individual degradation factor are provided in Supplementary Section 5 (Figs. S3–S6). The reconstruction quality is evaluated using four metrics: PSNR, Structural Similarity Index Measure (SSIM), Multi-Scale Structural Similarity Index Measure (MS-SSIM), and Learned Perceptual Image Patch Similarity (LPIPS). PSNR quantifies pixel-wise fidelity, while SSIM and MS-SSIM assess structural similarity at local and multi-scale levels, respectively. LPIPS evaluates semantic-level similarity by computing feature differences extracted from a convolutional neural network pretrained on a large-scale image dataset (AlexNet on ImageNet)^37^. Lower LPIPS values indicate better perceptual similarity. These metrics provide a comprehensive evaluation that reflects both numerical accuracy and semantic consistency of the reconstructed images. From the simulation results, the proposed method consistently outperforms the comparative methods across all the evaluation metrics and degradation levels. Notably, the performance gap becomes more pronounced under severe degradation conditions, highlighting our method’s robustness and generalization capability.

**Fig. 4:**
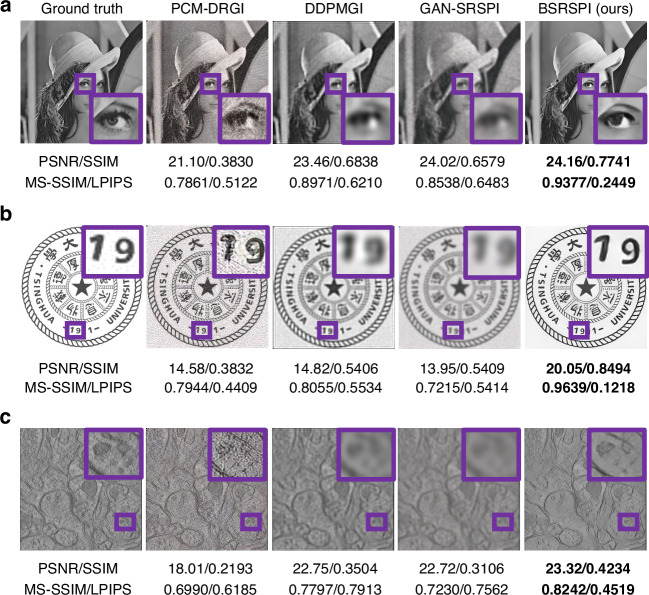
**Simulation results under composite degradation models.** The comparison includes PCM-DRGI^33^, DDPMGI^32^, and GAN-SRSPI^34^. Three representative cases are shown: **a** Set14^39^ No.009, **b** Tsinghua University logo, **c** Neuron in the molecular layer of a mouse cerebellum^41^. Their corresponding composite degradation settings and sampling rates are: **a** level 2 degradation, 5% sampling; **b** level 2 degradation, 6% sampling; and (c) level 2 degradation, 6.25% sampling. BSRSPI achieves the best perceptual quality across all conditions

Composite degradations are applied to images from Set5^38^ and Set14^39^ datasets to simulate complex real-world conditions. An image from the Set5 datasets is used to visualize representative reconstruction results, as shown in Fig. 4a. The model is also tested on the Tsinghua University logo, with the result shown in Fig. 4b. Additionally, to verify the model’s generalization capability, several images from the biomedical field are tested: a mitochondrion in a rod cell of a mouse retina^40^, a neuron in the molecular layer of a mouse cerebellum^41^, and the immunological synapse between a human cytotoxic T lymphocyte and a target cell^42^. The cerebellar neuron result is shown in Fig. 4c, with two additional examples provided in Supplementary Section 6, Fig. S8. It is noteworthy that these biomedical images are not in the dataset used for training. In this domain shift test, our method consistently achieves the best reconstruction results across all metrics and test samples. This generalization performance is attributed to the incorporation of the physical degradation model of single-pixel imaging, which enables the network to learn degradation-robust representations.

### Experimental results

**Fig. 5:**
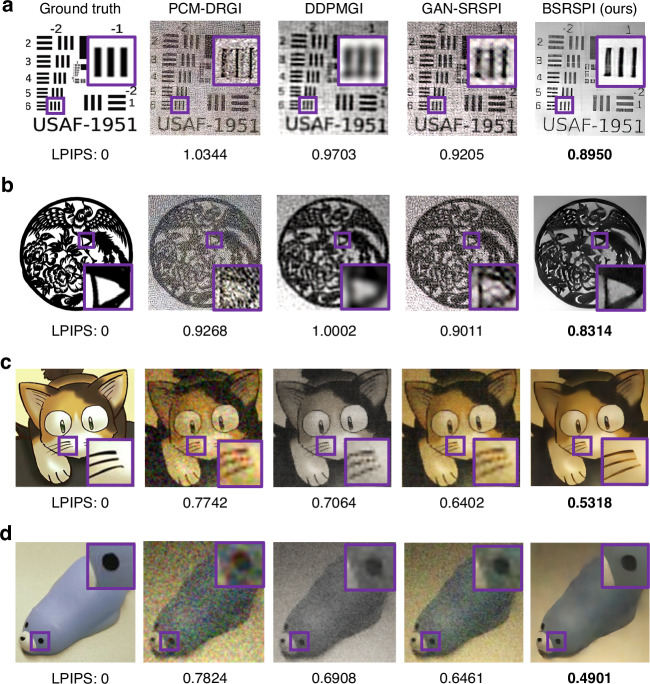
**Reconstruction results under combinations of real-world degradations, including mist-induced scattering, platform jitter, signal-dependent noise, and subsampling (sampling ratio: 6.25%).** **a**, **b** Are grayscale scenes under varying degradation levels, while **c**, **d** show color reconstructions. LPIPS is Learned Perceptual Image Patch Similarity, which evaluates the consistency of high-level semantic features; lower values indicate better semantic similarity between images

The performance of different reconstruction methods, including PCM-DRGI^33^, DDPMGI^32^, and GAN-SRSPI^34^, is evaluated on real-world degraded scenes in Fig. 5. All the tested samples are experimental single-pixel images collected under realistic conditions involving mist, jitter, and sensor noise. Reconstruction results are compared using LPIPS, a full-reference evaluation metric that evaluates image similarity in the deep feature space of a pretrained network. As shown in Fig. 5, the proposed method consistently achieves the lowest LPIPS scores across all samples, demonstrating superior semantic-level reconstruction quality under complex real-world degradations.

As shown in Fig. 5, experimental comparisons on the color image reconstruction performance of reconstruction methods PCM-DRGI^33^, DDPMGI^32^, GAN-SRSPI^34^, and the proposed BSRSPI are conducted on simulated color SPI data. The color measurements are generated using Hadamard-Bayer illumination patterns, which spatially encode RGB channels within a single pattern to enable full-color acquisition^43^. To ensure fairness and consistency with the experimental encoding scheme, all compared neural networks are fine-tuned under the corresponding physical model. Quantitative evaluation based on the LPIPS metric further confirms this advantage, with our proposed BSRSPI consistently obtaining the lowest LPIPS values, indicating greater perceptual consistency with the ground truth in terms of high-level image features. Additional experimental results are provided in Supplementary Section 6 (Fig. S9). The framework also supports varying levels of prior knowledge. In our experiment, the network operates in a fully blind setting that has no knowledge of the degradation type. The network can also be fine-tuned for better image reconstruction quality if the parameters of the degradation sources are known.

## Discussion

This work proposes a comprehensive degradation model for SPI. The model systematically integrates multiple physical degradation factors, such as downsampling, jitter, scattering, and readout noise. Two SPI degradation mechanisms are proposed: a pattern-dependent global noise propagation model that describes the non-ideal intensity variations in structured illumination, and a jitter-aware multiplicative noise model that accounts for the relative motion between the object and the projection system. Based on this structured degradation formulation, a deep-blind reconstruction network is trained to compensate for complex degradation combinations without requiring prior knowledge of degradation parameters. Experimental validation on both simulated and real-world data demonstrates that the proposed method consistently outperforms existing approaches across a wide range of degradation levels, confirming its robustness and effectiveness in practical SPI scenarios.

The proposed method serves as a general-purpose framework for modeling complex, compound degradations in real-world imaging systems. Its modular design allows degradation components to be inserted or adjusted based on application-specific requirements. For example, photobleaching or nonlinear scattering effects can be incorporated into the degradation model to compensate for artifacts that are difficult to parameterize and often lack aligned high-resolution ground truth. Beyond biomedical imaging, the method is adaptable to tasks such as remote sensing, surveillance, or industrial inspection, where unknown degradations commonly arise. By learning from a wide variety of simulated degradation patterns, the model maintains strong generalization capacity during deployment, even without large-scale retraining. These attributes highlight the framework’s practical value across diverse imaging scenarios.

## Materials and methods

### Experimental setup and real-world test

The experimental setup for verifying the reconstructed image quality of SPI is illustrated in Fig. 2a. A photograph of the actual experimental setup is provided in Supplementary Section 6, Fig. S7. The projector (JMGO G7) projects Hadamard basis illumination patterns arranged in the order of cake-cutting^25^ onto the printed target object. Simultaneously, the single-pixel detector (Thorlabs DET100A2) records the light intensity data of the reflected light. The analog output from the single-pixel detector is digitized using a digital oscilloscope (Rigol DS7014) and transmitted to a computer for image reconstruction. In the experiments, three types of real-world degradation are introduced in the optical path: noise, blur, and jitter. Noise is added to the collected single-pixel intensity signals to simulate sensor and photon-related fluctuations. Blur is induced by placing an ultrasonic humidifier (Jisulife HU18) between the optical system and the object to create a thin, scattering mist layer. Ultrasonic humidifiers are often used to generate micrometer-scale water droplets that mimic the optical degradation caused by fog, haze, or thin condensation layers in recent studies^44–46^. Jitter is introduced by manually perturbing the object position during acquisition to simulate mechanical instability or object movement in real-world scenarios. Moreover, recent advances in computational imaging highlight that deep learning provides powerful tools to model and compensate for such degradations^47^.

### Network training details

All training and inference procedures are implemented in PyTorch 2.4.1 (cu121) with CUDA 12.1 and executed on a Windows 10 workstation equipped with an NVIDIA RTX 3090 GPU. The proposed model is trained for 100,000 iterations. Our average inference time per 128 × 128 image is ~60 milliseconds. For the proposed model, the hyperparameters of the loss function are as follows: ${\alpha }_{{\rm{img}}}=1,{{\alpha }}_{{\rm{per}}}=0.1,{{\alpha }}_{{\rm{adv}}}=0.1$. These values are selected based on an ablation study discussed in Supplementary Section 7.

## Supplementary information

Supplementary Information

## Data Availability

The data that support the findings of this study are available from the corresponding author upon reasonable request.
