# Supplementary material for: Comprehensive compensation of real-world degradations for robust single-pixel imaging
Source: Light Sci Appl. 2025 Oct 13;14:365. doi: 10.1038/s41377-025-02021-7 (PMC12518823; doi:10.1038/s41377-025-02021-7)
Supplement: Supplementary file 1 — Supplementary Information [file 41377_2025_2021_MOESM1_ESM.docx]

**Supplementary Information for**

**Comprehensive compensation of real-world degradations for robust single-pixel imaging**

Zonghao Liu,^a,†^ Bohan Yang,^a,b^ Yifei Zhang,^a^ Junfei Shen,^c^ Xin Yuan,^d^ Mu Ku Chen,^e,*^ Fei Liu,^f,*^ Zihan Geng^a,b,*,†^

aTsinghua Shenzhen International Graduate School, Tsinghua University, Shenzhen 518055, China

bPengcheng Laboratory, Shenzhen 518055, China

cCollege of Electronics and Information Engineering, Sichuan University, Chengdu 610065, China

dAI Department, School of Engineering, Westlake University, Hangzhou 310030, China

eDepartment of Electrical Engineering, City University of Hong Kong, Kowloon, Hong Kong SAR 999077, China

fSchool of Optoelectronic Engineering, Xidian University, Xi'an 710071, China

†These authors contributed equally: Zonghao Liu, Zihan Geng.

*****Mu Ku Chen**,** E-mail: [mkchen@cityu.edu.hk](mailto:mkchen@cityu.edu.hk)

*****Fei Liu**,** E-mail: [feiliu@xidian.edu.cn](mailto:feiliu@xidian.edu.cn)

*****Zihan Geng**,** E-mail: [geng.zihan@sz.tsinghua.edu.cn](mailto:geng.zihan@sz.tsinghua.edu.cn)

**Section 1: Comparison of Conventional Imaging and Single Pixel Imaging (SPI) degradation Model**

**Section 2: Computational Ghost Imaging (CGI) Reconstruction**

**Section 3: Network Structure**

**Section 4: Data Generation**

**Section 5: Performance under Different Degradation Conditions**

**Section 6: Experimental Setup and Additional Results**

**Section 7: Ablation Study on Loss Function**

# Comparison of Conventional Imaging and Single Pixel Imaging (SPI) Degradation Models

Compared to conventional imaging, single-pixel imaging (SPI) involves a more elaborate and structured degradation process, reflecting its fundamentally different image formation pathway. Compared with conventional imaging, SPI includes many more operations. These include illumination pattern modulation, spatial integration across the scene, and pattern-dependent noise propagation.

In conventional imaging systems, degradation is often modeled as a linear sequence: optical blur introduced by the lens, followed by sensor-level sampling (downsampling), and finally additive sensor noise. In contrast, SPI operates in a fundamentally different regime. The scene is first modulated by structured illumination patterns, then the modulated light undergoes optical and detection-path degradation before being integrated into a single scalar measurement. This integration step introduces a global, non-local behavior that tightly couples spatial degradation with temporal and pattern-dependent noise sources.

Another key difference lies in how noise propagates. In conventional imaging, additive noise typically affects each pixel within a small local region. In SPI, each scalar measurement reflects the global response of the scene. Consequently, any noise in the measurement propagates across the entire reconstructed image, weighted by the structure of that pattern. This gives rise to a pattern-weighted global noise structure, which cannot be effectively modeled by conventional pixel-wise degradation frameworks.

These characteristics underscore the necessity of a physically faithful and task-specific degradation model for SPI. The following sections provide a structured comparison of the two paradigms, including detailed mathematical formulations of the degradation components and their interactions.


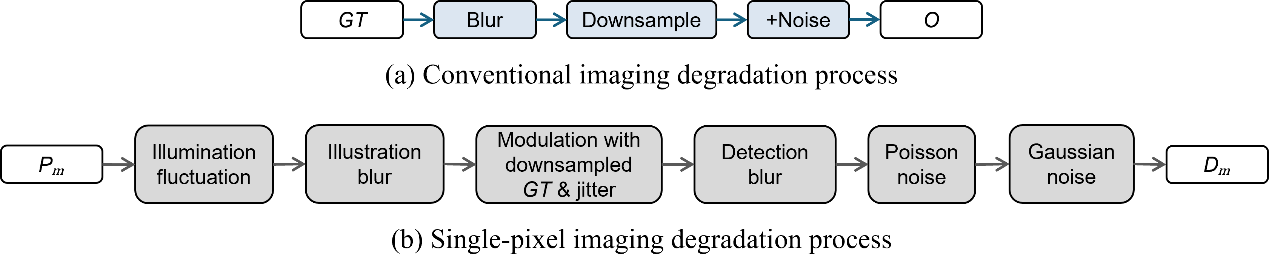


Fig. S1 Comparison of degradation processes in (a) conventional imaging and (b) single-pixel imaging (SPI). SPI includes additional physical steps such as illumination fluctuation, dual-path blurring, spatial integration, and pattern-dependent noise propagation.

## 1.1 Conventional Imaging Degradation

An image ground truth ($GT)$ is blurred by a kernel $k$, downsampled by a factor $s$, and finally corrupted by additive noise $n$, often modeled as Gaussian white noise. A degraded image O can be modeled as^1,2^:

|  | $O=\left( GT*k \right)\downarrow_{s}+n$ | (S1) |
| --- | --- | --- |

where:

- *GT*: original high-resolution image;
- *k*: blurring kernel (e.g., Gaussian);
- $\downarrow_{s}$​: uniform downsampling by factor *s*;
- *n*: additive noise, typically modeled as i.i.d. Gaussian noise $\mathcal{N}\left( 0,\sigma^{2} \right)$.

## 1.2 Degradation Order and Noise Propagation of SPI

The difference in degradation sequences between conventional imaging and SPI is schematically illustrated in Fig. S1a and Fig. S1b, respectively.

In SPI, the degradation and measurement process are reordered as:

|  | $P_{m}\to{\varepsilon_{illum,m}\cdot P}_{m}\left( x \right)\to\mathcal{B}_{1}\left( \cdot\right)\to{\downarrow_{s}\left( GT\left( x \right) \right)\cdot P}_{m}\left( x \right)$  $\to\mathcal{B}_{2}\left( \cdot\right)\to\text{integration}\to\cdot\left( 1+\delta_{m} \right)\to+\varepsilon_{add,m}\to D_{m}$ | (S2) |
| --- | --- | --- |

This formulation captures the complete degradation and measurement sequence in SPI. The original pattern $P_{m}$ first experiences multiplicative illumination fluctuation $\varepsilon_{illum,m}$, followed by illumination-path blurring $\mathcal{B}_{1}\left( \cdot\right)$. The ground truth scene $GT\left( x \right)$ is then spatially downsampled in a block-averaged manner ($\downarrow_{s}$) and pointwise multiplied with the blurred illumination pattern, the resulting product passed through detection-path blurring $\mathcal{B}_{2}\left( \cdot\right)$, and finally subjected to global spatial integration as part of the single-pixel measurement process. After integration, pattern-dependent multiplicative noise ($1+\delta_{m}$) is applied, followed by additive Gaussian noise $\varepsilon_{m}$ at the measurement stage.

Each step corresponds to a distinct physical process: $\varepsilon_{illum,m}$ models fluctuations in illumination intensity; $\mathcal{B}_{1}\left( \cdot\right)$ and $\mathcal{B}_{2}\left( \cdot\right)$ capture scattering and blur before and after modulation, respectively; $\downarrow_{s}$ simulates pixel-level averaging under subsampling; and $\delta_{m}$ accounts for spatial jitter modulated by the boundary complexity of illumination patterns; $\varepsilon_{\mathrm{add},m}$ represents both the Poisson noise due to photon arrival statistics and the additive Gaussian noise introduced during the measurement stage. The integration step, unique to SPI, performs a global operation that aggregates modulated intensities across the entire scene.

This non-commutative structure implies that the output is fundamentally altered when the order of operations is changed^3^. In particular, the spatial integration that occurs after modulation in SPI is a nonlinear global operation—it cannot be interchanged with spatial-domain degradations such as blurring or noise addition. Similarly, multiplicative noise (e.g., from jitter or illumination fluctuation) and convolutional blur do not generally commute due to their nonlinear interaction^3^. Consequently, degradation modeling in SPI must preserve the actual physical order of operations. This limits the effectiveness of directly applying conventional degradation models to SPI and highlights the necessity of a dedicated framework as proposed in this work.

## 1.3 Pattern-dependent Noise Propagation of SPI

In the idealized linear model of SPI, the reconstructed image can be mathematically expressed as:

|  | $O\left( x \right)=\sum_{i=1}^{N} D_{m}\cdot P_{m}\left( x \right)$ | (S3) |
| --- | --- | --- |

Hence, a measurement perturbation $\delta D_{m}$​ propagates as:

|  | $\delta O\left( x \right)=\delta D_{m}\cdot P_{m}\left( x \right)$ | (S4) |
| --- | --- | --- |

Here, $x$ is the location of each pixel, and $D_{m}$ is the measurement result of the *m*-th pattern. Since patterns $P_{m}\left( x \right)$ (e.g., Hadamard, random, Fourier) are global or semi-global, localized noise in the measurement space propagates across the entire image domain. This effect is referred to as pattern-weighted global noise, wherein the structured modulation distributes measurement noise non-uniformly in the reconstruction.

This behavior stands in stark contrast to conventional imaging systems, where additive noise typically affects only local neighborhoods in the image domain. In conventional imaging, the observed image $O\left( x \right)$ can be expressed as:

|  | $O\left( x \right)=GT\left( x \right)+n\left( x \right),\quad n\left( x \right)\sim\mathcal{N}\left( 0,\sigma^{2} \right)$ | (S5) |
| --- | --- | --- |

where $GT\left( x \right)$ is the ground-truth image and $n\left( x \right)\sim\mathcal{N}\left( 0,\sigma^{2} \right)$ denotes zero-mean Gaussian noise independently added at each pixel. The noise $n\left( x \right)$ is spatially uncorrelated and localized, meaning that perturbations at one pixel do not propagate across the image. This pixel-wise additive structure contrasts with the pattern-weighted global noise in SPI.

In SPI, however, each measurement perturbation spreads globally according to its associated pattern. As a result, the noise structure in SPI is inherently coupled with the modulation basis, reinforcing the necessity for degradation modeling approaches that explicitly account for this pattern-weighted global propagation.

# Computational Ghost Imaging (CGI) Reconstruction

As the first stage of the reconstruction pipeline, the classical Computational Ghost Imaging (CGI) method is applied to obtain a coarse image from the raw single-pixel measurements. Its mathematical formulation is detailed below. The recovered image $O\left( x,y \right)$ is obtained by correlating the known illumination patterns with the measured intensity signals as:

$\begin{matrix} O\left( x,y \right)=\frac{1}{M}\sum_{m=1}^{M} \left( D_{m}-\hat{D} \right)\left[ P_{m}\left( x,y \right)-\hat{P}\left( x,y \right) \right] \end{matrix}$ (S6)

where *O* symbolizes the image obtained post-reconstruction, while (*x,y*) specifies the pixel coordinates within the reconstructed image. *M* denotes the number of illumination patterns utilized during the reconstruction process, with *m* representing the index of a particular measurement iteration. The acquired light intensity signal is indicated by *D*, whereas $\hat{\ldots}$ stands for the average value, and *P* corresponds to the illumination pattern itself. The underlying physics behind this equation hinges on the correlation between the known illumination patterns and their respective single-pixel detector intensity measurements. Leveraging this correlation enables the effective reconstruction of the target scene.

# Network Structure

To address the inherent challenges of SPI, an image super-resolution and blind degradation compensation network (Fig. S2) is developed and trained by the physical degradation model, in order to overcome globally coupled degradation, signal-dependent noise, and extreme underdetermination under low sampling ratios in real-world SPI imaging.

First, a multi-scale discriminator with hierarchical downsampling and skip-connected upsampling is employed to capture both large-scale structural consistency and localized texture fidelity. This design reflects the non-local nature of degradation in SPI, where each measurement corresponds to a global projection of the illumination pattern onto the entire scene. As a result, noise introduced by a single pattern propagates across the entire reconstruction, in contrast to conventional imaging systems where degradation tends to be locally confined. The proposed architecture enhances the network’s ability to suppress globally modulated noise and pattern-dependent artifacts, thereby improving robustness to real-world degradation factors in SPI.

Second, perceptual supervision is incorporated to provide high-level semantic guidance consistent with natural image distributions. Under extremely low sampling conditions, pixel-wise loss alone often results in over-smoothed reconstructions with missing high-frequency components. The perceptual loss evaluates similarity in a deep feature space and encourages the reconstruction of structurally plausible textures, edges, and contours. Within the proposed framework, statistical priors from natural images, physical priors derived from the SPI forward model, and direct measurement data are jointly leveraged. This integration mitigates the intrinsic trade-off between sampling time and reconstruction quality, enabling perceptually faithful recovery from sparse measurements.

The overall architecture consists of a generator and a multi-scale discriminator, designed to support high-fidelity reconstruction under complex degradations. The generator begins with a shallow convolutional front-end, followed by 23 residual-in-residual dense blocks, each comprising three densely connected dense blocks with five convolutional layers and ReLU activations per block. This structure preserves both low-level detail and high-level semantic features while avoiding normalization layers to maintain local contrast. Two upsampling stages—each combining nearest-neighbor interpolation and convolution—progressively increase the resolution by a factor of four, producing the super-resolved output.

The discriminator features a symmetric downsampling and upsampling structure, consisting of three convolutional layers (stride 2) for hierarchical downsampling, followed by three bilinear upsampling stages, each fused with skip connections from the corresponding encoder layers. All intermediate layers, except the first and final ones, are regularized with spectral normalization. This design enables multi-resolution feature integration and provides spatially-aware adversarial supervision. It enhances sensitivity to both local structural distortions and global consistency deviations arising from pattern-coupled degradation effects in SPI reconstruction.

In contrast to the conventional MSE-based loss functions^4,5^, a more sophisticated loss function^6^ is used in this framework, which caters to a wider array of objectives and yields superior outcomes

|  | $L_{\mathrm{Gen}}=\alpha_{\mathrm{img}}L_{\mathrm{img}}+\alpha_{\mathrm{adv}}L_{\mathrm{adv}}+\alpha_{\mathrm{per}}L_{\mathrm{per}}$ | (S7) |
| --- | --- | --- |

Here, *L*_Gen_ denotes the generator network's loss function, while *L*_img_, *L*_adv_, and *L*_per_ represent the image loss, adversarial loss, and perceptual loss, respectively. $\alpha_{\mathrm{img}}$, $\alpha_{\mathrm{adv}}$, and $\alpha_{\mathrm{per}}$ are hyperparameters.

The image loss is defined as the L1 distance between the generated output and the ground truth image. It encourages pixel-wise fidelity to the original image

|  | $L_{\text{img}}=\frac{1}{N}\sum_{i=1}^{N} \left\vert I_{i,\text{HR}}-\left( G\left( I_{\text{LR}} \right) \right)_{i} \right\vert$ | (S8) |
| --- | --- | --- |

In this equation, $N$ denotes the total number of pixels, and $i$ indexes each pixel. $I_{i,\text{HR}}$​ is the $i$-th pixel of the high-resolution ground-truth image, $I_{\text{LR}}$​ is the low-resolution input image, and $G\left( \cdot\right)$ is the generator mapping $I_{\text{LR}}$​ to the reconstructed super-resolution image.


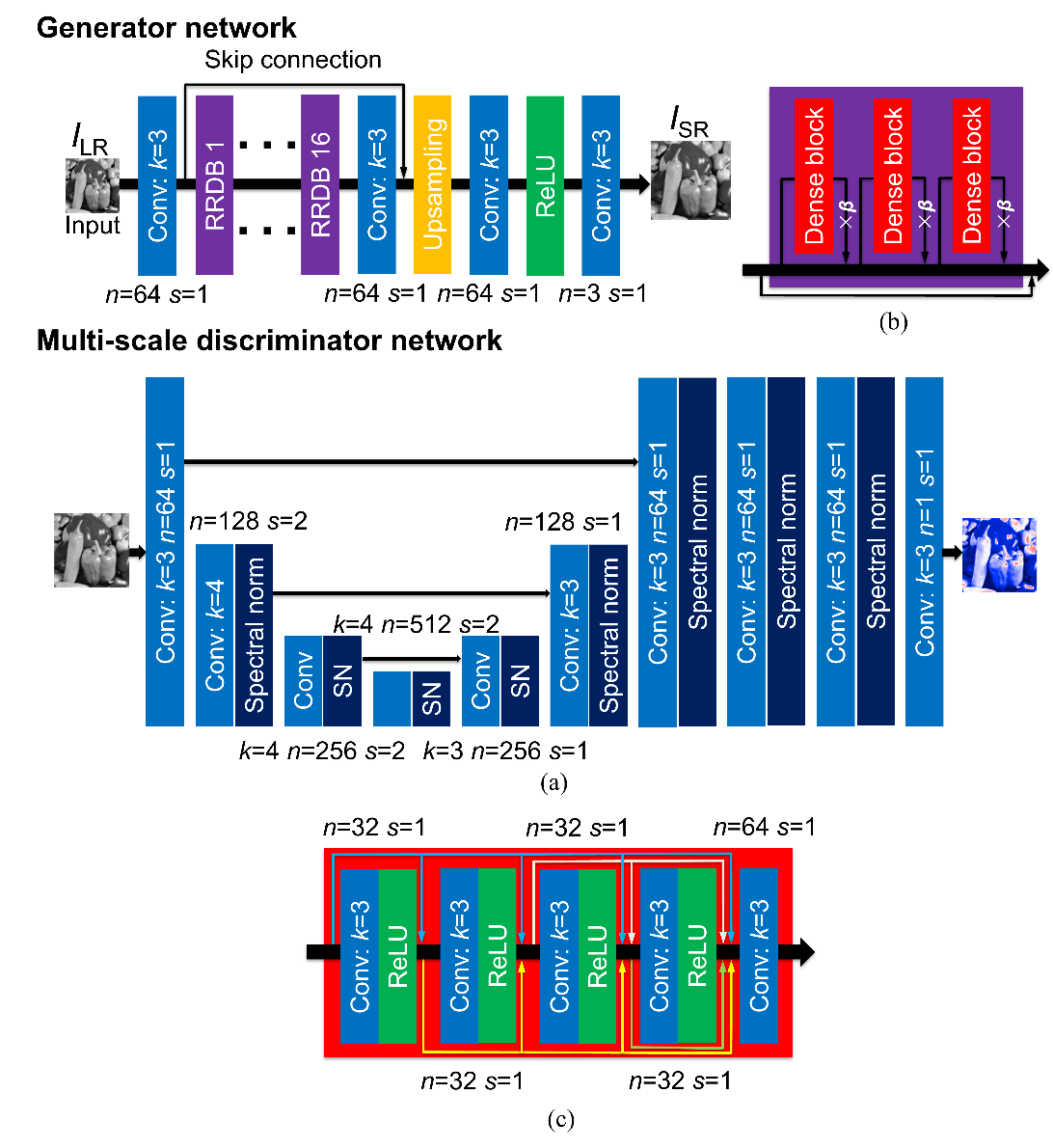


Fig. S2 Schematic diagram of the neural network structure of BSRSPI, where *k* denotes the convolutional kernel size, *n* denotes the number of feature mappings, and *s* denotes the step size. (a) Network structure of the generator and the multi-scale discriminator. (b) Structure of the residual-in-residual dense block. (c) Structure of the dense block.

The adversarial loss characterizes the ability of the generator to produce images that deceive the discriminator, i.e., causing the discriminator to struggle in differentiating between the generated and real images

|  | $L_{\text{adv}}=\frac{1}{N\cdot H\cdot W}\sum_{n=1}^{N} \sum_{i=1}^{H} \sum_{j=1}^{W} \left( D\left( G\left( I_{n,L\text{R}} \right) \right)_{i,j}-1 \right)^{2}$ | (S9) |
| --- | --- | --- |

In this expression, $N$ denotes the batch size, and $H\cdot W$ is the spatial size of the discriminator’s output map. The term $D\left( G\left( I_{n,L\text{R}} \right) \right)_{i,j}$​ represents the pixel-level realness score predicted by the discriminator $D$ at location ($i,j$) for the super-resolved image generated by generator $G$ from the $n$-th low-resolution input. This adversarial loss encourages the generator to produce outputs that are indistinguishable from real images across all spatial locations. The discriminator’s loss is formulated symmetrically, using target values of 1 for real images and 0 for fake ones.

The perceptual loss employs features within the Visual Geometry Group (VGG)^7^ neural network to compute the L1 distance between the generated image and the target image

|  | $L_{\mathrm{per}}=\sum_{k=1}^{K} w_{k}\cdot\frac{1}{N_{k}}\sum_{j=1}^{N_{k}} \left\vert\phi_{k}\left( I_{H\text{R}} \right)_{j}-\phi_{k}\left( G\left( I_{L\text{R}} \right) \right)_{j} \right\vert$ | (S10) |
| --- | --- | --- |

In this formula, $\phi_{k}$ denotes the *k*-th feature map extracted by the VGG network, *K* represents the total number of feature maps used for computation, $N_{k}$ signifies the total number of pixels within the *k*-th feature map, and *j* corresponds to the respective pixels. $I_{\text{HR}}$​ is the high-resolution ground-truth image, $I_{\text{LR}}$​ is the low-resolution input image, and $G\left( \cdot\right)$ is the generator mapping $I_{\text{LR}}$​ to the reconstructed super-resolution image.

To supervise the reconstruction process, pixel-wise image loss is combined with perceptual loss. The image loss $L_{\mathrm{img}}$ is defined as the L1 distance between the reconstructed image and the ground truth at the pixel level. While effective for enforcing strict structural alignment, this loss tends to produce over-smoothed results and suppress high-frequency textures—especially under severe or blind degradations—since it penalizes all pixel deviations equally, regardless of perceptual importance^8-10^.

To alleviate this limitation, a perceptual loss $L_{\mathrm{per}}$ is introduced, which compares high-level feature representations between the output and the reference image. These features are extracted from a VGG19 network pretrained on the ImageNet^11-13^ dataset. The network is originally trained for large-scale image classification over 1000 categories using more than 1.2 million natural images. As a result, its intermediate activations capture semantically meaningful and perceptually relevant structures, beyond low-level pixel differences^14^.

In this study, the features from VGG19 layers—relu1_1, relu2_1, relu3_3, relu4_3, and relu5_4 are used. Here, relu*X*_*Y* denotes the activation output from the *Y*-th convolutional layer in the *X*-th VGG19 block. These layers contain basic texture features and high-level semantics. The selected layers are empirically weighted as [0.1, 0.1, 1.0, 1.0, 1.0] to emphasize mid- and high-level feature. By jointly optimizing $L_{\mathrm{img}}$ and $L_{\mathrm{per}}$, the network is guided to produce reconstructions that are not only numerically accurate but also visually natural and consistent with human perception.

# Data Generation

To improve generalization and realistically reflect the signal formation process in real-world SPI, a structured stochastic degradation strategy is introduced. This strategy captures the compound nature of SPI degradations and serves as the basis for training the reconstruction network. Unlike conventional degradation pipelines with fixed sequences, the proposed strategy samples degradation components independently based on predefined probabilities and applies them in a physically meaningful order.

The physical degradation model of SPI is followed, specifically in Eq. (1) of the main text, where each SPI measurement *Dₘ* is formulated as a spatial integration of a physically degraded scene under structured illumination. These equations explicitly model the sequential degradations including illumination fluctuation, blurring, spatial integration, and measurement noise. In the context of training data generation, stochastic sampling of degradation components with randomized parameters is performed, while preserving the physically consistent structure of the forward SPI model. Additionally, a sampling ratio *r* ∈ [0.05, 0.0625] is introduced to control the proportion of Hadamard patterns used in each simulation.

## 4.1 Structured Degradation Framework

To generate realistic and diverse training data for blind SPI reconstruction, a structured stochastic degradation strategy that reflects the actual physical imaging process is adopted. This strategy introduces probabilistic variation in degradation types, strengths, and combinations, while preserving the physically consistent order defined in the forward model.

Specifically, each degradation component—such as illumination noise, blurring, jitter, and additive measurement noise—is independently sampled with a predefined probability (see Table S1). For example, illumination blur is applied in 80% of cases, while multiplicative jitter appears in 20% of samples. The “Probability” column in Table S1 thus indicates how likely each degradation is activated in a given training instance.

Compared to fixed or sequential degradation pipelines, this probabilistic formulation enables broader coverage of realistic degradation combinations. As demonstrated in the experiments, it significantly improves the model’s robustness to unseen noise configurations and enhances generalization to real-world settings.

## 4.2 Physically Motivated Degradation Components

The degradation components are:

- **Illumination multiplicative noise**: $\varepsilon_{\mathrm{illum},m}$ ~ 𝒩(1, $\sigma_{\mathrm{illum}}$²), with $\sigma_{\mathrm{illum}}$ sampled from [0.02, 0.2]. This noise is first applied multiplicatively to the illumination pattern $P_{m}$, simulating intensity fluctuations from the light source or spatial modulator.
- **Illumination blur**: Gaussian blur applied to the illumination pattern $P_{m}$ before modulation, either isotropic ($\sigma_{\mathrm{iso},m}$ ∈ [0.2, 2.0]) or anisotropic $\sigma_{x,m}$, $\sigma_{y,m}$ ∈ [0.2, 2.0]).
- **Block-averaged downsampling**: Reduction of image resolution by averaging non-overlapping blocks of size $s \times s$ (with $s = 4$), effectively implementing spatial downsampling consistent with Hadamard-based SPI.
- **Detection blur**: Gaussian blur applied after modulation, with the same sampling range as illumination blur.
- **Multiplicative jitter noise**: Applied after spatial integration as $\delta_{m}$ ~ 𝒩(0, $\sigma_{\text{jitter}\text{,}\text{m}}^{2}$), with $\sigma_{\text{jitter}\text{,}\text{m}}^{2}$ ∈ [0.2, 2.0] ×$B_{m}$ scaled by the boundary complexity of the illumination pattern. Here, $B_{m}$​ denotes the normalized boundary length of the $m$-th illumination pattern, reflecting the spatial complexity of the pattern edges. The jitter noise variance $\sigma_{\text{jitter}\text{,}\text{m}}^{2}$ scales proportionally with $B_{m}$​, capturing how more complex pattern boundaries induce greater measurement perturbations.
- **Additive Gaussian noise**: $\varepsilon_{\mathrm{add},m}$ ~ 𝒩(0, $\sigma_{\text{add}\text{,}\text{m}}^{2}$), with $\sigma_{\text{add}\text{,}\text{m}}^{2}=\sigma_{P\text{,}\text{m}}^{2}+\sigma_{\mathrm{AG}}^{2}$. The photon-related noise term $\sigma_{P\text{,m}}^{2}$​ approximates Poisson-distributed shot noise and is modeled as $\sigma_{P\text{,}\text{m}}^{2}$ ∈ [0.001, 0.01] ×$D_{\mathrm{norm},m}$, where $D_{\mathrm{norm},m}$​ denotes the normalized signal intensity corresponding to the $m$-th pattern. The term $\sigma_{AG}$ ∈ [1.0, 10.0] represents signal-independent electronic noise and models the final measurement noise floor.

Each component is activated with a predefined sampling probability. Specifically, illumination noise, illumination blur, and detection blur are applied with 80% probability; additive Gaussian noise with 95%; and multiplicative jitter noise with 50%. Block-averaged downsampling is always applied. For the blur components, when activated, there is an equal probability of applying isotropic or anisotropic Gaussian blur. In the anisotropic case, the standard deviations satisfy $\sigma_{x,m}=\sigma_{y,m}$​, effectively reducing to an isotropic blur.

## 4.3 Sampling Configuration and Parameter Summary

The degradation configuration is randomly generated per training image. Table S1 summarizes the components:

Table S1 Modular degradation components in model training.

| **Component** | **Symbol** | **Sampling Range** | **Probability** | **Application Order** |
| --- | --- | --- | --- | --- |
| Illumination noise | $\varepsilon_{\mathrm{illum},m}$ | $\sigma_{\mathrm{illum}}$ ∈ [0.02, 0.2] | 80% | First stage |
| Illumination blur | $\mathcal{B}_{1}\left( \cdot\right)$ | $\sigma_{x,m}$, $\sigma_{y,m}$ ∈ [0.2, 2.0] | 80% | Before modulation |
| Downsampling | $\downarrow_{s}\left( \cdot\right)$ | *s* = 4 (fixed) | Always | Always |
| Detection blur | $\mathcal{B}_{2}\left( \cdot\right)$ | Same as illumination blur | 80% | After modulation |
| Multiplicative jitter noise | $\delta_{m}$ | $\sigma_{\text{jitter,}\text{m}}^{2}$ ∈ [0.2, 2.0] ×$B_{m}$ | 50% | After integration |
| Additive Gaussian noise | $\varepsilon_{\mathrm{add},m}$ | $\sigma_{P\text{,}\text{m}}^{2}$∈[0.001, 0.01] ×$D_{\mathrm{norm},m}$  $\sigma_{\mathrm{AG}}$ ∈ [1.0, 10.0] | 95% | Final stage |
| Sampling ratio | $r$ | *r* ∈ [0.05, 0.0625] | 100% | Global parameter |

## 4.4 Representative Degradation Examples

To demonstrate the stochastic degradation pipeline, one complete degradation path and two simplified variants are illustrated below:

- **Path 1 (Full degradation chain, sampling ratio r = 0.0625):** illumination noise $\varepsilon_{\mathrm{illum}}$ ($\sigma_{\mathrm{illum}}$ = 0.05) → illumination blur *B*₁ (isotropic, $\sigma_{\mathrm{iso},m}$ ∈ [0.2, 2.0]) → spatial downsampling ↓*ₛ* → detection blur *B*₂ (anisotropic, $\sigma_{x,m}$, $\sigma_{y,m}$ ∈ [0.2, 2.0]) → modulation jitter $\delta$($\sigma_{\text{jitter}\text{,}\text{m}}^{2}$= 0.5 × $B_{m}$) → Poisson-Gaussian noise $\varepsilon_{\mathrm{add}}$ ($\sigma_{\mathrm{AG}}$ = 10.0, $\sigma_{P\text{,}\text{m}}^{2}$= 0.01 × $D_{\mathrm{norm},m}$)
- **Path 2 (Reduced version, sampling ratio r = 0.06):** illumination noise $\varepsilon_{\mathrm{illum}}$ ($\sigma_{\mathrm{illum}}$ = 0.08) → illumination blur *B*₁ (anisotropic, $\sigma_{x,m}$, $\sigma_{y,m}$ ∈ [0.2, 2.0]) → spatial downsampling ↓*ₛ* → detection blur *B*₂ (isotropic, $\sigma_{\mathrm{iso},m}$ ∈ [0.2, 2.0]) → Poisson-Gaussian noise $\varepsilon_{\mathrm{add}}$ ($\sigma_{\mathrm{AG}}$ = 2.5, $\sigma_{P\text{,}\text{m}}^{2}$= 0.001 × $D_{\mathrm{norm},m}$)
- **Path 3 (Reduced version, sampling ratio r = 0.05):** illumination blur *B*₁ (anisotropic, $\sigma_{x,m}$, $\sigma_{y,m}$ ∈ [0.2, 2.0]) → spatial downsampling ↓*ₛ* → modulation jitter $\delta$($\sigma_{\text{jitter}\text{,}\text{m}}^{2}$= 0.5 × $B_{m}$) → Poisson-Gaussian noise $\varepsilon_{\mathrm{add}}$ ($\sigma_{\mathrm{AG}}$ = 7.0, $\sigma_{P\text{,}\text{m}}^{2}$= 0.004 × $D_{\mathrm{norm},m}$)

The degraded measurements are reconstructed using the SPI reconstruction formula (S6).

# Performance under Different Degradation Conditions

To verify the effectiveness and robustness of the proposed blind degradation model, reconstruction performance is evaluated on a pre-segregated test dataset under four representative degradation types commonly encountered in single-pixel imaging systems. These include:

(i) **Multiplicative illumination noise**, modeled as independent Gaussian perturbations applied to each projected pattern to simulate frame-to-frame fluctuations in light source intensity and DMD modulation fidelity. Ten levels of severity are defined, with the standard deviation $\sigma_{\mathrm{illum}}$ ranging from 0.02 to 0.2;

(ii) **Scattering-induced spatial blur**, simulated using Gaussian kernels in both isotropic and anisotropic forms. Blur severity is controlled by adjusting the kernel width $\sigma_{x,m}$, $\sigma_{y,m}$ ∈ [0.2, 2.0] from 0.2 to 2.0;

(iii) **Random jitter**, implemented by randomly shifting the measurement window around the target patch within a larger high-resolution scene. For each illumination pattern, a displaced subregion is extracted to simulate the effect of relative motion between the projection system and the object. The severity level is defined as the probability of applying displacement, increasing from 10% to 100% across ten levels;

(iv) **Additive detection noise**, comprising signal-dependent Poisson noise and signal-independent Gaussian readout noise. The Poisson noise variance $\sigma_{P\text{,}\text{m}}^{2}$​ is defined as [0.001, 0.01] × $D_{\mathrm{norm},m}$​, discretized into ten uniform levels. Likewise, the Gaussian noise standard deviation $\sigma_{AG}$​ is set across ten levels from 1.0 to 10.0.

For composite degradation evaluation, all the degradation types are applied simultaneously at the same severity level. That is, Level n composite degradation includes illumination noise, blur, jitter, and additive noise all set at their respective Level n configurations.

Comparative methods include three representative deep learning-based approaches that reflect diverse network architectures and reconstruction strategies for ghost imaging or single-pixel imaging: PCM-DRGI (Photon Contribution Model-based Degradation-Guided Ghost Imaging)^15^, which adopts a dual-branch CNN guided by estimated photon degradation, where photon count maps inform spatially adaptive reconstruction. DDPMGI (Denoising Diffusion Probabilistic Model Ghost Imaging)^16^, a generative restoration framework based on denoising diffusion modeling, which progressively refines image quality through an iterative reverse process. GAN-SRSPI (GAN-based Super-Resolution Single-Pixel Imaging)^17^, a GAN-based model using residual blocks in the generator and a PatchGAN discriminator, which enforces local perceptual realism by evaluating small image patches rather than the entire image. These methods differ in architectural design and in how they implicitly or explicitly address measurement degradation. They are used as comparative baselines in both simulation and experimental evaluations. In contrast, the proposed method integrates a physically grounded and structured degradation model into a blind reconstruction framework, enabling robust generalization across diverse and uncharacterized degradation scenarios.

For each degradation type, its severity is progressively increased while the other degradation sources are fixed at baseline values, allowing for isolated analysis of its individual impact. Reconstruction quality is assessed using four standard metrics: PSNR, SSIM, MS-SSIM, and LPIPS (higher is better for the first three, lower is better for LPIPS).

The results, presented in Supplementary Figs. S3–S6, show that the proposed BSRSPI consistently outperforms competing methods across a wide range of degradation levels in all four scenarios.


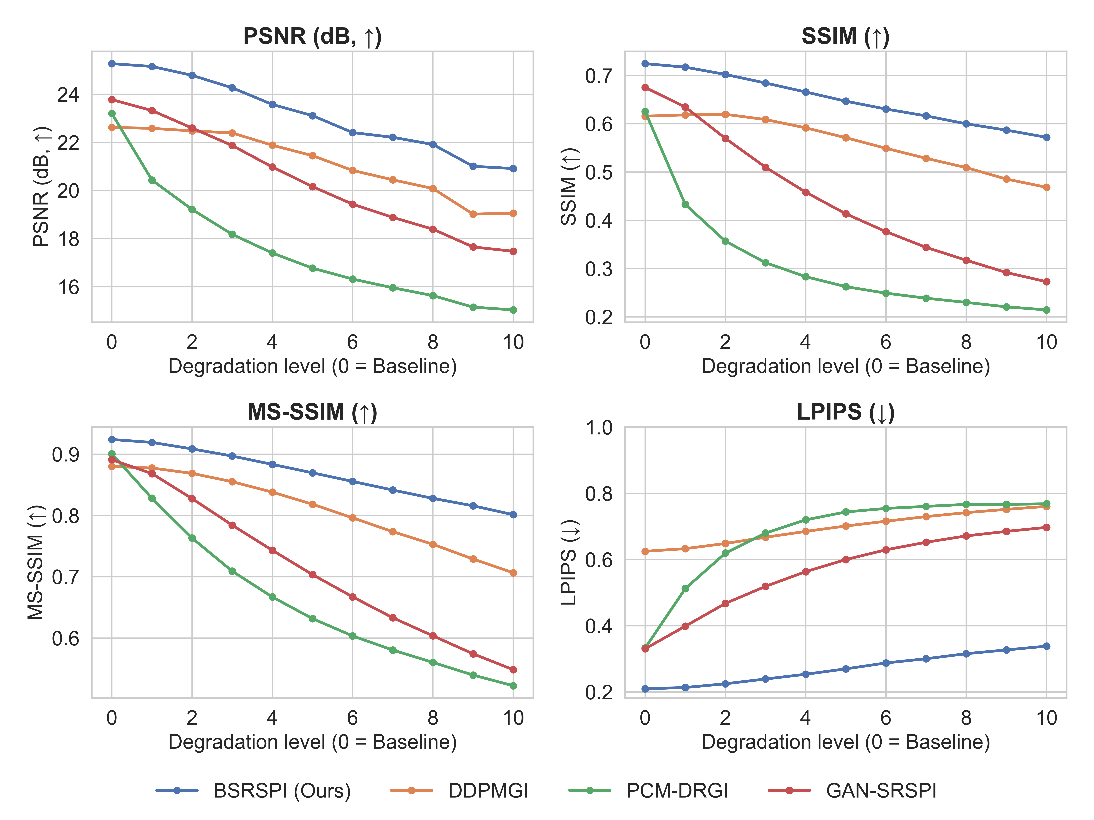


Fig. S3 Impact of additive detection noise on reconstruction performance. Evaluation of BSRSPI and competing methods under increasing levels of additive noise, including Poisson noise and Gaussian readout noise. Other degradation sources are fixed at baseline levels. Metrics include PSNR (↑), SSIM (↑), MS-SSIM (↑), and LPIPS (↓). BSRSPI demonstrates superior robustness across all noise levels.


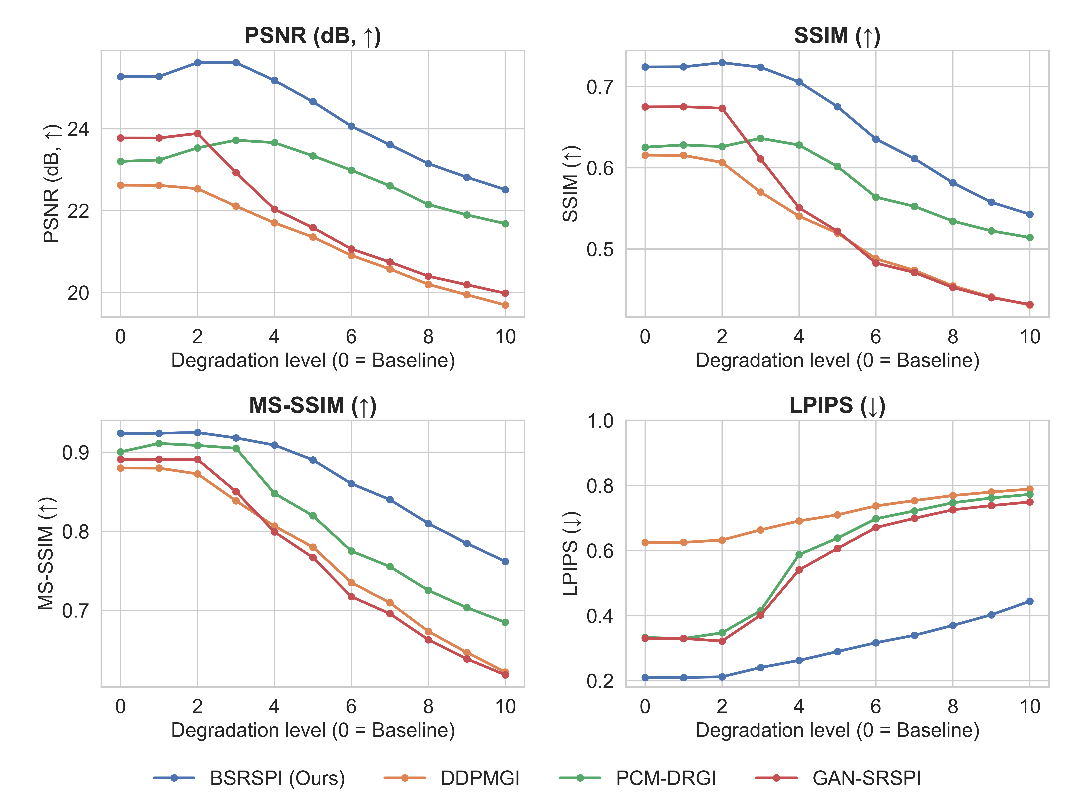


Fig. S4 Impact of scattering-induced blur on reconstruction performance. Performance comparison under progressively increasing blur strength, introduced along both the illumination and detection paths. Blur is modeled using isotropic and anisotropic Gaussian kernels. All other degradation components are held constant. BSRSPI consistently outperforms alternatives across all metrics and blur intensities.


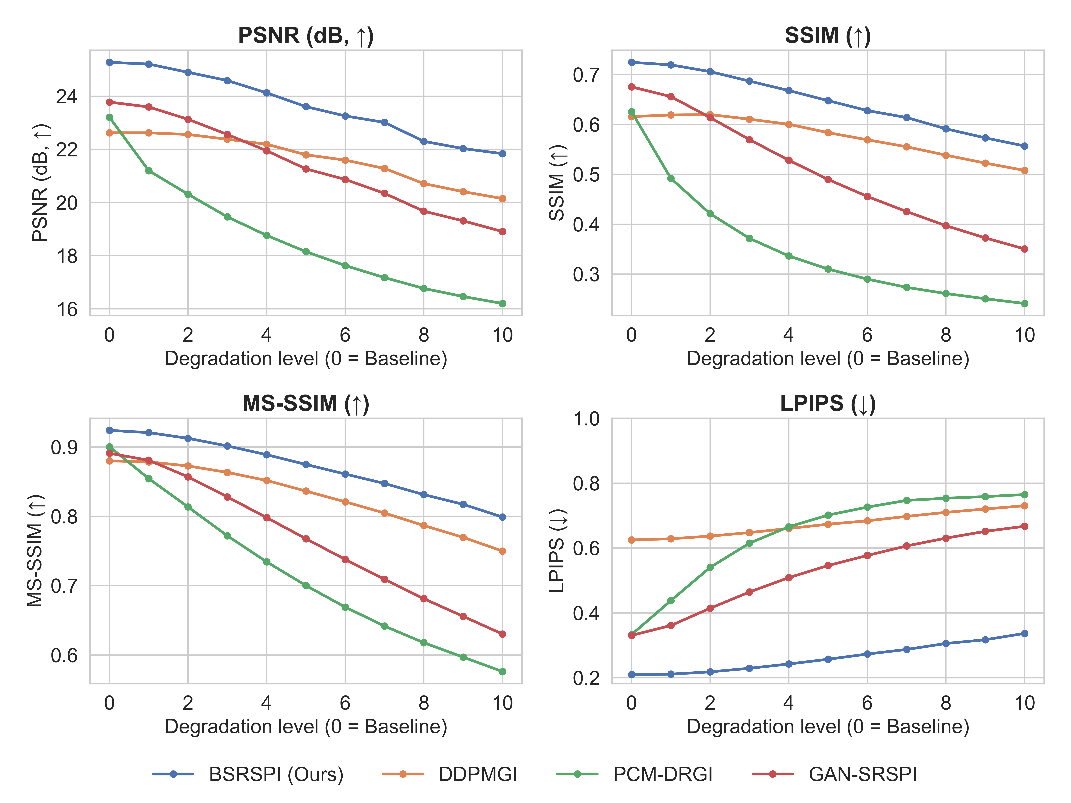


Fig. S5 Impact of spatial jitter on reconstruction performance. Spatial jitter is simulated by randomly shifting the ground-truth patch within a larger scene window, mimicking acquisition misalignment due to system instability. Jitter severity increases from 10% to 100%, while other degradation types remain fixed. BSRSPI consistently preserves reconstruction fidelity across all jitter levels, demonstrating strong robustness to spatial displacement.


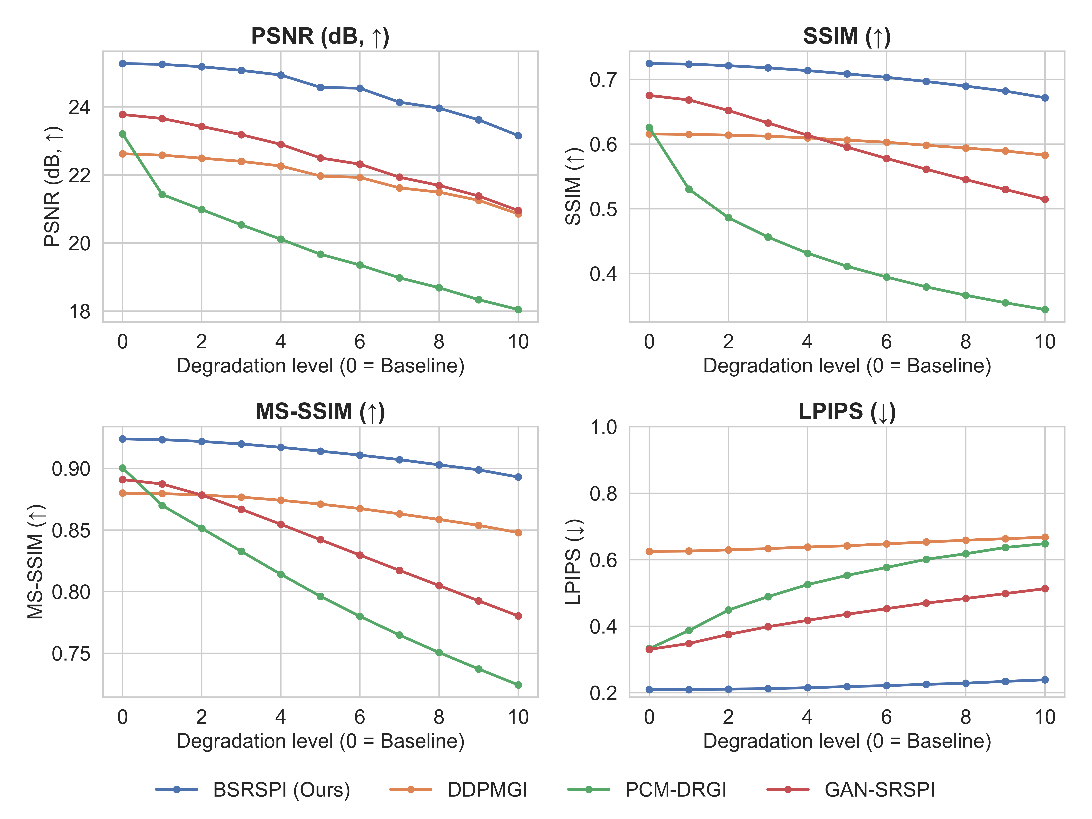


Fig. S6 Impact of multiplicative illumination noise on reconstruction performance. Analysis of performance under increasing levels of multiplicative fluctuations in illumination intensity, applied on a per-pattern basis. All other degradations are maintained at baseline. BSRSPI maintains perceptual and structural integrity better than baseline methods.

# Experimental Setup and Additional Results

To demonstrate the applicability of the proposed method in real-world scenarios, a SPI system is constructed that incorporates multiple sources of physical degradation. As illustrated in Fig. S7, the system includes a projector for structured illumination, a photodetector for signal integration, and a digital oscilloscope for acquisition. Degradation factors are introduced through a combination of hardware and environmental settings: blur caused by mist scattering (via an ultrasonic humidifier) and spatial misalignment due to platform instability. The schematic diagram (Fig. S7a) outlines the full signal flow of the SPI pipeline, while the photograph (Fig. S7b) highlights the spatial location of each degradation source.

To validate reconstruction performance under compound degradation settings, extended simulation results in Fig. S8 are provided, which include examples from natural images (Set5) and biological microscopy datasets. Each example is subjected to a specific degradation level. Across all test cases, the proposed BSRSPI method demonstrates visually superior reconstruction quality compared to existing methods such as PCM-DRGI, DDPMGI, and GAN-SRSPI, especially in retaining fine structures and semantic details under multiple degradations.


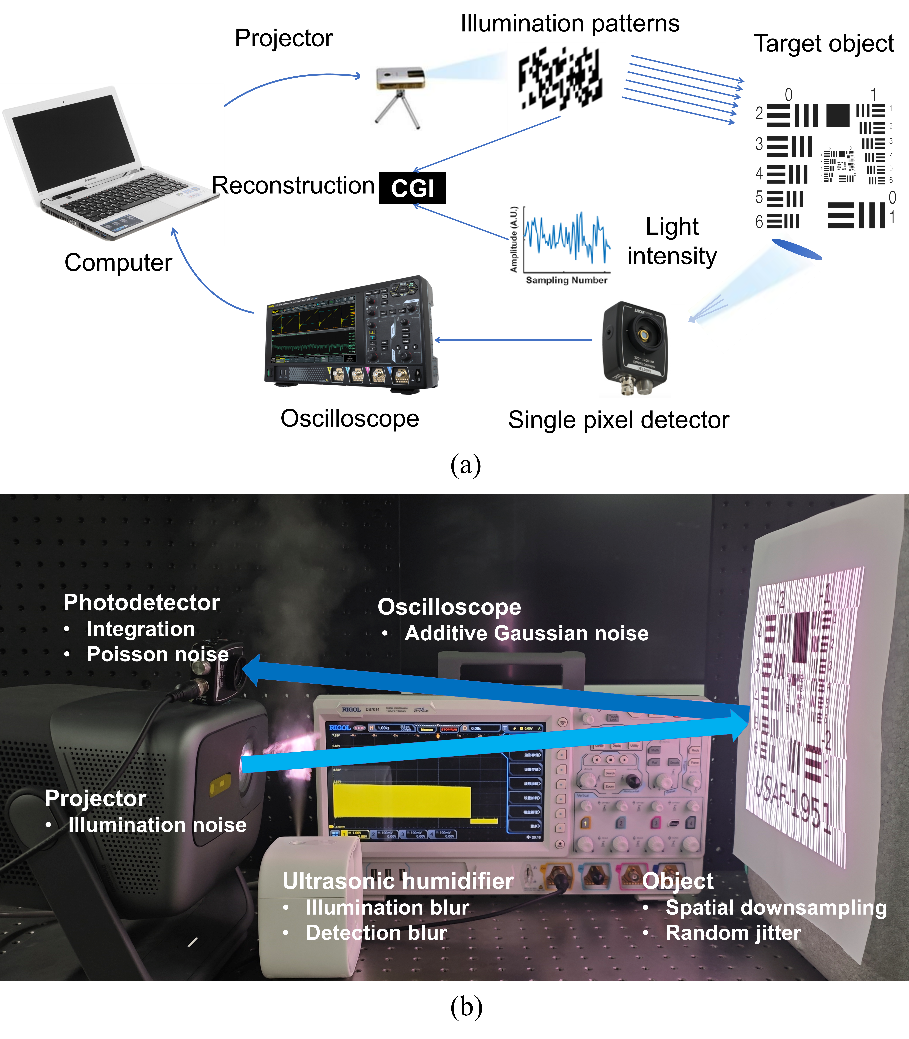


Fig. S7 Schematic and physical setup of the single-pixel imaging system with real-world degradations. (a) Schematic diagram illustrating the optical path and signal flow in the single-pixel imaging system, including pattern projection, photodetection, and signal acquisition via CGI reconstruction. (b) Photograph of the experimental setup, with annotations highlighting the physical sources of degradation: illumination noise from the projector, scattering-induced blur from the ultrasonic humidifier, additive Gaussian noise from the oscilloscope, and spatial downsampling and jitter from object instability.


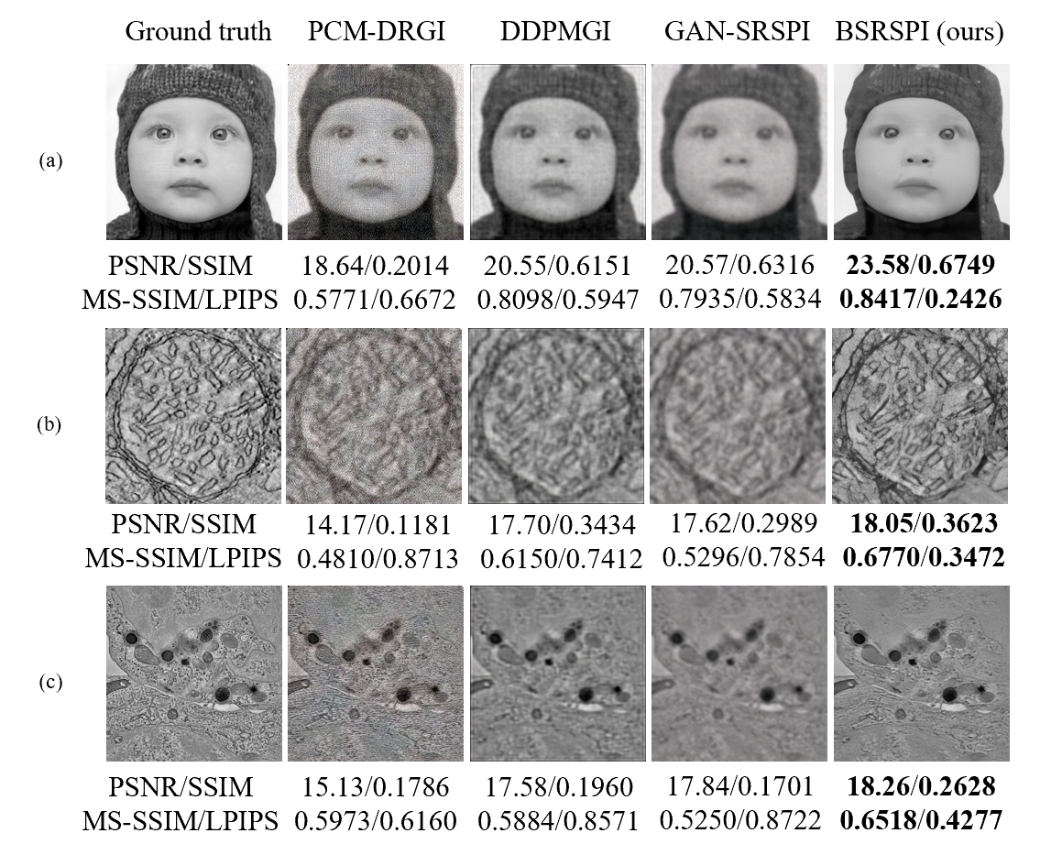


Fig. S8 Extended simulation results under compound degradation settings. The selected test images include: (a) Set5^18^ #001, (b) a mitochondrion in a rod cell of a mouse retina^19^, and (c) an immunological synapse between a human cytotoxic T lymphocyte and a target cell^20^. The corresponding composite degradation levels and sampling rates are: (a) level 4 / 6.25%, (b) level 5 / 6.25%, and (c) level 3 / 6.25%. The comparison includes PCM-DRGI^15^, DDPMGI^16^, GAN-SRSPI^17^, and the proposed BSRSPI, which consistently demonstrates superior perceptual quality across diverse content types and degradation conditions.


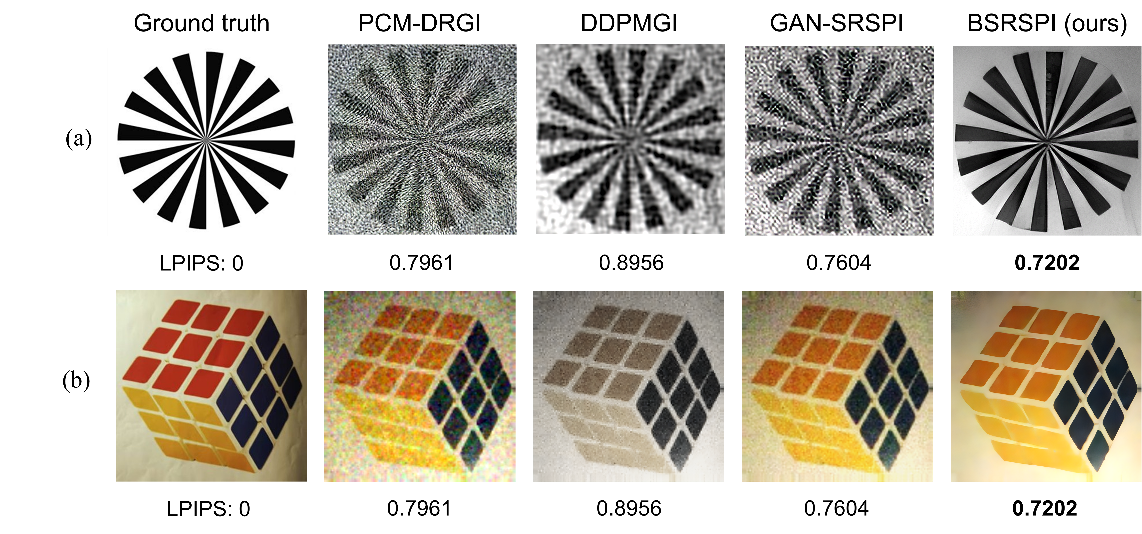


Fig. S9 Additional optical experimental results under combined real-world degradations. (a) Grayscale resolution target under noise level 10, 2% mist-induced scattering, 2% platform jitter, and a 5% sampling rate. (b) Color image of a third-order Rubik’s Cube, captured under combined degradations. In both cases, the proposed BSRSPI method demonstrates superior robustness in reconstructing fine structures and texture under complex real-world conditions.

In Fig. S9, additional optical experimental results under real-world combined degradations are presented to validate the method's robustness in practical settings. The test images include a high-frequency grayscale resolution target and a color image of a Rubik’s Cube, both acquired at strong degradation conditions including noise, mist-induced blur, and spatial jitter. In both cases, BSRSPI achieves noticeably better reconstruction fidelity and robustness compared to other methods, confirming its generalization ability and practical value in uncontrolled imaging environments.

# Ablation Study on Loss Function

To investigate the impact of different loss terms on the reconstruction quality, a lightweight ablation study is conducted by varying the weights assigned to image loss $\alpha_{\mathrm{img}}$, perceptual loss $\alpha_{\mathrm{per}}$, and adversarial loss $\alpha_{\mathrm{adv}}$ in the generator's objective function. A total of 13 combinations are evaluated, including single-loss settings, dual-loss configurations, and full combinations with varied relative weights.

Each model variant is trained for 10000 iterations under identical conditions, using the same network architecture, optimizer, training data, and batch size as the main experiments. Reconstruction quality is evaluated on a synthetic test set with multiple real-world degradation factors, including blur, illumination noise, subsampling, and signal-dependent measurement noise. PSNR and SSIM are used as evaluation metrics.

The results are summarized in Table S2. The highest overall performance is achieved with the loss configuration ${(\alpha}_{\mathrm{img}}$, $\alpha_{\mathrm{per}}$, $\alpha_{\mathrm{adv}}$) = (1.0, 0.1, 0.1), yielding the best PSNR (22.63 dB) and a near-optimal SSIM (0.6647). This configuration is adopted as the final setting due to its superior balance between quantitative fidelity and perceptual quality. In comparison, the baseline model trained with image loss alone (1.0, /, /) shows lower performance across all metrics. These results indicate that incorporating perceptual and adversarial losses not only improves visual realism, but also enhances structural fidelity. Such improvements can be attributed to the semantic priors introduced by the perceptual features and the regularization effect of the adversarial objective, which together steer the generator away from over-smoothed solutions and promote globally consistent reconstructions.

Table S2 Ablation study on loss function weights. Each model is evaluated on the synthetic test set with multiple real-world degradations.

| Config | $\alpha_{\mathrm{img}}$ | $\alpha_{\mathrm{per}}$ | $\alpha_{\mathrm{adv}}$ | PSNR (dB) (↑) | SSIM (↑) |
| --- | --- | --- | --- | --- | --- |
| 1 | 1.0 | / | / | 22.09 | 0.6313 |
| 2 | 1.0 | / | 0.1 | 21.50 | 0.6222 |
| 3 | 1.0 | 0.1 | 1 | 22.39 | 0.6586 |
| 4 | 1.0 | 0.1 | / | 21.48 | 0.6484 |
| 5 | / | 0.1 | / | 8.42 | 0.2977 |
| 6 | / | 0.1 | 0.1 | 15.13 | 0.5457 |
| 7 | / | / | 0.1 | 6.68 | 0.2997 |
| 8 | 10.0 | 0.1 | 0.1 | 22.05 | 0.6327 |
| 9 | 0.1 | 0.1 | 0.1 | 21.86 | 0.6529 |
| 10 | 1.0 | 1.0 | 0.1 | 20.49 | 0.5889 |
| 11 | 1.0 | 0.01 | 0.1 | 20.28 | 0.5754 |
| 12 | 1.0 | 0.1 | 0.01 | 22.59 | 0.6645 |
| 13 | 1.0 | 0.1 | 0.1 | **22.63** | **0.6647** |

Furthermore, as long as the pixel-wise image loss is included, the network maintains reasonably good performance across a wide range of weight combinations. The addition of perceptual and adversarial losses provides further improvements in both PSNR and SSIM, though the gains are relatively moderate. In contrast, removing the image loss entirely results in severe degradation, with unstable reconstructions and notably lower fidelity. This highlights that the pixel loss plays a central role in maintaining structural integrity, while the perceptual and adversarial losses serve to refine texture and perceptual quality. Together, they form a complementary and robust loss formulation.

Importantly, this behavior is particularly beneficial in the context of single-pixel imaging, where the reconstruction task is highly ill-posed due to extreme under-sampling. The combined use of perceptual and adversarial priors helps compensate for missing spatial information, yielding structurally consistent and perceptually plausible reconstructions. Based on these findings, the configuration ${(\alpha}_{\mathrm{img}}$, $\alpha_{\mathrm{per}}$, $\alpha_{\mathrm{adv}}$) = (1.0, 0.1, 0.1) is selected as the default setting throughout all experiments reported in this work.

## References

1. Liu, C. & Sun, D. On Bayesian adaptive video super resolution. *IEEE Trans. Pattern Anal. Mach. Intell.* **36**, 346-360 (2014).

2. Shocher, A., Cohen, N. & Irani, M. “zero-shot” super-resolution using deep internal learning. In Proc. of the IEEE Conference on Computer Vision and Pattern Recognition, 3118-3126 (IEEE, Salt Lake City, 2018).

3. Zhang, K., Liang, J., Van Gool, L. & Timofte, R. Designing a practical degradation model for deep blind image super-resolution. In Proc. of the IEEE/CVF International Conference on Computer Vision, 4791-4800 (IEEE, 2021).

4. Dong, C., Loy, C. C., He, K. & Tang, X. Image Super-Resolution Using Deep Convolutional Networks. *IEEE Trans. Pattern Anal. Mach. Intell.* **38**, 295-307 (2016).

5. Shi, W. et al. Real-time single image and video super-resolution using an efficient sub-pixel convolutional neural network. In Proc. of the IEEE Conference on Computer Vision and Pattern Recognition, 1874-1883 (IEEE, Las Vegas, 2016).

6. Bruna, J., Sprechmann, P. & LeCun, Y. Super-resolution with deep convolutional sufficient statistics, Preprint at <https://arxiv.org/abs/1511.05666> (2015).

7. Simonyan, K. & Zisserman, A. Very deep convolutional networks for large-scale image recognition, Preprint at <https://arxiv.org/abs/1409.1556> (2014).

8. Wang, Z., Simoncelli, E. P. & Bovik, A. C. Multiscale structural similarity for image quality assessment. In Proc. of the Asilomar Conference on Signals, Systems & Computers, 1398-1402 (IEEE, Pacific Grove, 2003).

9. Wang, Z., Bovik, A. C., Sheikh, H. R. & Simoncelli, E. P. Image quality assessment: From error visibility to structural similarity. *IEEE Trans. Image Process.* **13**, 600-612 (2004).

10. Gupta, P., Srivastava, P., Bhardwaj, S. & Bhateja, V. A modified PSNR metric based on HVS for quality assessment of color images. In Proc. of the International Conference on Communication and Industrial Application, 1-4 (IEEE, Kolkata, 2011).

11. Deng, J. et al. Imagenet: A large-scale hierarchical image database. In Proc. of the IEEE Conference on Computer Vision and Pattern Recognition, 248-255 (IEEE, Miami Beach, 2009).

12. Ledig, C. et al. Photo-realistic single image super-resolution using a generative adversarial network. In Proc. of the IEEE Conference on Computer Vision and Pattern Recognition, 4681-4690 (IEEE, Honolulu, 2017).

13. Johnson, J., Alahi, A. & Fei-Fei, L. Perceptual losses for real-time style transfer and super-resolution. In Proc. fo the European Conference on Computer Vision, 694-711 (Springer, Amsterdam, 2016).

14. Ferwerda, J. A. Three varieties of realism in computer graphics. In Proc. of the Human Vision and Electronic Imaging, 290-297 (SPIE, Santa Clara, 2003).

15. Gao, Z., Cheng, X., Yue, J. & Hao, Q. Extendible ghost imaging with high reconstruction quality in strong scattering medium. *Opt. Express* **30**, 45759-45775 (2022).

16. Mao, S. et al. High-quality and high-diversity conditionally generative ghost imaging based on denoising diffusion probabilistic model. *Opt. Express* **31**, 25104-25116 (2023).

17. Liu, Z. et al. GAN-SRSPI: super-resolution single-pixel imaging using generative adversarial networks. In Proc. of the 9th Symposium on Novel Photoelectronic Detection Technology and Applications, 2009-2013 (SPIE, Hefei, 2022).

18. Bevilacqua, M., Roumy, A., Guillemot, C. & Alberi-Morel, M. L. Low-complexity single-image super-resolution based on nonnegative neighbor embedding. In Proc. of the British Machine Vision Conference, 1–10 (BMVA, Surrey, 2012).

19. Fox, D. & Perkins, G. CCDB:54, mus musculus, mitochondrion, photoreceptor/cone. Cell Centered Database (CCDB). <https://doi.org/10.7295/W9CCDB54> (2001).

20. Fuller, S., Majorovits, E., Griffiths, G., Stinchcombe, J. & Bossi, G. CCDB:3632, Homo sapiens, CTL immunological synapse, Cytotoxic T Lymphocyte. Cell Centered Database (CCDB). <https://doi.org/10.7295/W9CCDB3632> (2004).
